# Longitudinal inconsistency in responses to survey items that ask women about intimate partner violence

**DOI:** 10.1186/s12874-019-0835-4

**Published:** 2019-10-29

**Authors:** Deborah Loxton, Jennifer Powers, Natalie Townsend, Melissa L. Harris, Peta Forder

**Affiliations:** Research Centre for Generational Health and Ageing, School of Medicine and Public Health, University of Newcastle, Callaghan, New South Wales Australia

**Keywords:** Intimate partner violence, Longitudinal studies, Cohort studies, Surveys and questionnaires, Self report, Response consistency, Reliability

## Abstract

**Background:**

Longitudinal research has demonstrated that experiences of abuse and adversity are not consistently reported over time; however, there is a paucity of available evidence regarding the consistency of reporting experiences of intimate partner violence (IPV) over time. This study aimed to investigate the consistency of self-reported IPV involving a partner or spouse. Differences in the health status of women according to consistency of IPV reporting was also investigated.

**Methods:**

Survey data collected from the 1946–51 cohort of the Australian Longitudinal Study on Women’s Health (ALSWH) between 1996 and 2016 were used (*n* = 13,715). Consistency of self-reported IPV was evaluated by responses to the question “Have you ever been in a violent relationship with a partner/spouse?” Demographic and health characteristics of consistent and inconsistent reporters of IPV were compared. Multinomial logistic regression was used to determine the strength of the association between demographic and health characteristics of the women and their consistency of longitudinal reporting of IPV.

**Results:**

There were 10,966 women who answered IPV questions over six surveys, with 9610 women (87.6%) providing consistent responses. Inconsistent responses were provided by 1356 women (12.4%), of whom 258 (2.4%) reported IPV at all but one survey (Mainly IPV), 587 (5.3%) reported no IPV at all but one survey (Mainly no IPV), and 511 (4.7%) reported Mixed IPV responses over time. Women in the Mainly IPV group, and those in the Mixed IPV group were similar to those in the Consistent IPV group in demographic and health characteristics, whereas women in the Mainly no IPV group were similar to those in the Consistent never IPV group.

**Conclusions:**

IPV data collected at one time point may involve around 12% false negative or false positive responses. To increase reliability, IPV should be measured on more than one occasion, using different techniques and methods that account for intentional and unintentional over- and under-reporting.

## Background

Intimate partner violence (IPV) is a significant public health problem. Experiences of IPV have been shown to be associated with a range of physical health problems, such as breathing difficulties, asthma, emphysema, bronchitis, allergies, fatigue, low iron, vaginal discharge, cervical cancer, hearing and eyesight problems, bowel problems [1], chronic disease [2], and chronic pain [2, 3]. Women who have experienced IPV are also more likely to have mental health issues, including depression [4–6], anxiety [4, 7, 8], suicidal ideation [2], and post-traumatic stress disorder [8, 9].

Globally, reported prevalence rates of IPV vary from 0.98 to 70.9% [3]. Research has demonstrated that prevalence rates differ depending on a number of methodological and cultural differences. Factors which affect IPV prevalence rates include the demographics of the sample (such as age and cultural background) [10, 11], the source of the sample (such as a clinical setting or nationally representative sample) [12, 13], the method (such as interviews or surveys) [14], and questions used to attain the information (such as asking participants to identify ‘violent’ experiences or specific behaviours) [15].

While methodological differences might explain some of this variation, one potential explanation that has received little attention is the consistency with which an individual might report IPV over time. Longitudinal research has demonstrated that participants do not consistently report abuse and experiences of adversity over time, with problems in consistency of self-reporting identified for experiences of physical abuse [16], sexual abuse [16, 17], potentially traumatic events [18], and negative life events [19]. Although there is evidence that experiences of abuse and adversity are not consistently reported over time, there is a paucity of available evidence regarding the consistency of reporting experiences of IPV over time.

The aim of this study is to investigate the consistency of self-report IPV involving a partner or spouse. The primary purpose is to identify and describe women who consistently reported IPV over 17 years, consistently reported later onset of IPV, consistently reported never experiencing IPV, and women who were inconsistent in their reports of IPV. Differences in the health status of women according to consistency of IPV reporting will also be investigated.

## Methods

### Sample

The current study examined IPV consistency using data collected from the 1946–51 cohort of the Australian Longitudinal Study on Women’s Health (ALSWH). The ALSWH cohorts have been described in detail elsewhere [20]. Briefly, women were randomly selected from the Medicare (National Health Insurance) database, (which covers all Australian citizens and permanent residents), with women from rural and remote areas intentionally oversampled at twice the rate as other women. The 1946–51 cohort were recruited via mailed surveys in 1996 (*n* = 13,715) and have completed follow-up surveys every 3 years since 1998. Comparisons between women of the same age in the 1996 Census showed the women were broadly representative of Australian women of the same age with some over-representation of tertiary educated women [21]. This study is approved by the Human Research Ethics Committees of the Universities of Newcastle and Queensland. Women were included in the current study if they had responded to the first survey in 1996 and at least two of the five subsequent surveys between 2004 and 2016.

### Measures

All variables were self-reported and were asked at each survey, unless otherwise indicated.

Consistency of self-report of IPV was evaluated by responses to the question, ‘Have you ever been in violent relationship with a partner/spouse?’ This question was first asked at Survey 1 in 1996 (*n* = 13,714) and at follow-up surveys conducted in 2004 (Survey 4, *n* = 10,905), 2007 (Survey 5, *n* = 10,638), 2010 (Survey 6, *n* = 10,011), 2013 (Survey 7, *n* = 9151), and 2016 (Survey 8, *n* = 8622). An affirmative response to this question was used to indicate IPV.

To determine consistency of IPV reporting over time, data on IPV were required from participants for at least three of the six surveys. Responses were classified as 'Consistent IPV' (i.e. IPV was reported at all surveys answered e.g. YYY, YYYYYY), 'Consistent later IPV' (i.e. did not experience IPV at earlier surveys but did at later surveys e.g. NYY, NNY, NNNNYY), 'Consistent never IPV' (i.e. never experienced IPV at any survey e.g. NNN, NNNNNN) and 'Inconsistent IPV' (i.e. provided a negative response after an affirmative response for IPV). This group was further split into “Mainly IPV” (i.e. reported IPV at all but one subsequent survey e.g. YNY, YYN, YYYNYY), 'Mainly no IPV' (i.e. reported IPV at one survey followed by no IPV for all other surveys e.g. YNN, NYN, YNNNNN) and 'Mixed IPV responses' (i.e. inconsistently reported IPV across time e.g. YNYN, YYN, YNNY, YNNNYN).

Area of residence was classified using the Accessibility/Remoteness Index of Australia (ARIA+), which measures accessibility to services from a person's home [22] and was grouped into four categories (major city, inner regional, outer regional and remote/very remote). In 1996, women were asked to indicate the highest qualification they had completed. Responses were categorised as ‘Less than Year 12’, ‘Year 12 or equivalent’, ‘Trade, Certificate or Diploma’, or ‘University degree or higher degree’. At each survey, relationship status was classed as ‘Married’, ‘De facto’ (cohabiting), ‘Separated’, ‘Divorced’, ‘Widowed’ or ‘Single’. Change in relationship status over time was classified in terms of being separated or divorced: never separated or divorced, remained separated or divorced, became partnered at a later survey, or became separated or divorced at a later survey.

At each survey, women were asked how stressed they felt about their relationship with their partner or spouse. Responses of ‘Very stressed’ or ‘Extremely stressed’ were used to indicate high partner stress. If participants indicated that they were very or extremely stressed about their relationship with their partner for at least half of their completed surveys, they were categorised as being highly stressed about their partner over time.

An indicator for experiencing financial stress is measured at each survey with the question “How do you manage on the income you have available?” Response options of ‘Impossible’ and ‘Difficult all the time’ were used to indicate high financial stress, while responses of ‘Easy’, ‘Not too bad’ and ‘Difficult some of the time’ suggested low financial stress. Participants were classified as being highly stressed about income over time if they indicated that they had high financial stress for at least half of their completed surveys.

### Statistical analyses

Demographic and health characteristics were compared for consistent and inconsistent reporters of IPV, with selected characteristics compared for baseline (1996) and over time. Multinomial logistic regression was used to determine the strength of associations between demographic and health characteristics of the women and their longitudinal reporting of IPV. These models simultaneously estimate the odds ratios and their 95% confidence intervals for the explanatory variables for each IPV group, relative to the Consistent IPV group (i.e. always reported IPV consistently). A sensitivity analysis was done with the multinomial logistic regression repeated for women who responded to the IPV questions at all surveys. All analyses were conducted in SAS version 9.4 [23] and statistical significance was set at 0.05.

## Results

Of the 10,966 women who answered IPV questions over six surveys, 2530 (23.1%) reported IPV at least once. Nearly two-thirds (65.1%) of women responded to all six surveys, and a further 26.4% responded to four or five surveys (Table 1). Consistent responses to IPV questions were given by 9610 women (87.6%), with 827 women consistently reporting IPV at all surveys (7.5%; Consistent IPV), 347 women consistently reporting IPV at later surveys (3.2%; Consistent later IPV) and 8436 consistently reporting they had never experienced IPV (76.9%; Consistent never IPV). Inconsistent responses were provided by 1356 women (12.4%), with 258 women reporting IPV at all but one survey (2.4%; Mainly IPV), 587 women reporting no IPV at all but one survey (5.4%; Mainly no IPV), and 511 women reporting mixed responses over time (4.7%; Mixed IPV). There were 2721 women who were not eligible for inclusion (ie. did not answer the IPV question at Survey 1 and at least two subsequent surveys), with most of these women (69%) responding to Survey 1 only. Women who were ineligible for this analysis reported a higher prevalence of IPV at Survey 1 (21.4%) than those who were eligible (15.0%).

**Table 1:** Proportion of women consistently and inconsistently reporting IPV over time (*N* = 10,966)

	Consistent IPV reporting			Inconsistent IPV reporting			
	Consistent IPV	Consistent later IPV	Consistent never IPV	Mainly IPV	Mainly no IPV	Mixed IPV	All women
Number of times IPV question answered	*N* = 827	*N* = 347	*N* = 8436	*N* = 258	*N* = 587	*N* = 511	*N* = 10,966
3 (*n* = 936)	13.0	15.6	7.9	15.9	12.3	0.0	8.5
4 (*n* = 1173)	14.2	17.3	9.9	11.2	14.3	9.2	10.7
5 (*n* = 1722)	15.7	19.0	15.2	20.9	16.5	17.8	15.7
6 (*n* = 7135)	57.1	48.1	67.0	51.9	56.9	73.0	65.1

Column percentages

Women who reported ‘Consistent never IPV’ or ‘Mainly no IPV’ were very similar in qualifications (Table 2). The ‘Consistent never IPV’ group of women were most likely to be married in 1996, least likely to be separated or divorced at any time, were least stressed about their partner and were least likely to be stressed about their income in 1996 or later. Women who reported ‘Consistent IPV’ or ‘Mainly IPV’ were most likely to be separated or divorced in 1996, to stay that way over time and to have financial difficulty. Women who had ‘Mixed IPV’ responses across surveys were similar to those women who consistently reported IPV (Consistent IPV) in regards to health and demographic variables.

**Table 2:** Characteristics of women according to consistency of reporting IPV over time

	Consistent IPV reporting			Inconsistent IPV reporting		
	Consistent IPV	Consistent later IPV	Consistent never IPV	Mainly IPV	Mainly no IPV	Mixed IPV
	*N* = 827	*N* = 347	*N* = 8436	*N* = 258	*N* = 587	*N* = 511
Area of residence – Survey 1 (%)						
Major city	64.8	69.4	67.3	62.5	63.3	66.0
Inner regional area	21.5	20.2	20.5	24.5	23.5	21.7
Outer regional area	11.1	8.0	10.1	10.8	11.4	10.1
Remote/very remote	2.6	2.4	2.0	2.1	1.8	2.2
Qualifications – Survey 1 (%)						
Less than Year 12	52.7	50.4	42.9	54.2	43.7	49.1
Year 12 or equivalent	15.7	18.6	17.3	15.9	18.1	15.5
Trade/Certificate/Diploma	19.0	17.4	20.9	18.0	20.1	17.8
University degree	12.6	13.6	18.9	11.9	18.1	17.6
Relationship status – Survey 1 (%)						
Married/Defacto	57.6	77.5	86.3	57.2	77.3	67.6
Divorced/Separated	36.9	17.4	8.1	33.5	17.1	29.1
Widowed/Single	5.5	5.1	5.6	9.3	5.6	3.2
Relationship status over surveys (%)						
Never divorced/separated	42.9	49.0	83.7	47.4	69.5	49.2
Remained divorced/separated	21.0	12.2	5.1	21.4	9.8	16.5
Became partnered	15.8	5.2	3.0	12.1	7.5	13.0
Became divorced/separated	20.2	33.6	8.2	19.1	13.2	21.3
Stressed about partner/spouse (%)						
At Survey 1	17.0	17.6	5.5	21.5	11.9	20.4
For at least half of surveys	4.4	7.7	1.5	7.3	2.9	5.8
Stressed about income (%)						
At Survey 1	27.5	23.7	10.2	27.6	19.0	20.8
For at least half of surveys	23.2	23.6	6.4	25.0	13.0	17.5

Percentages weighted for intentional oversampling of women living in rural and remote areas of Australia

There was little evidence to suggest that area of residence or highest qualification were associated with patterns of IPV reporting over time (Table 3). Women who remained separated/divorced or became separated/divorced were more likely to report no IPV at all surveys (Consistent never IPV) or no IPV at most surveys (Mainly no IPV) than women in the other groups. Women who were stressed about their partner over time were less likely to report no IPV at all surveys (Consistent never IPV), while women who were stressed about income over time were less likely to report IPV at all surveys (Consistent IPV) or mostly no IPV (Consistent no IPV) across surveys. Compared to women who completed six surveys, women who completed fewer surveys were more likely to report IPV consistently at later surveys (Consistent later IPV) or more likely to report IPV at most surveys (Mainly IPV).

**Table 3:** Characteristics of women by consistency of reporting IPV over time for women who responded to at least three surveys (*N* = 10,881)^a^

Characteristics	Consistent later IPV (OR, 95% CI)	Consistent never IPV (OR, 95% CI)	Mainly IPV (OR, 95% CI)	Mainly no IPV (OR, 95% CI)	Mixed IPV (OR, 95% CI)
Area of residence					
Major city	1	1	1	1	1
Inner regional area	0.85 (0.64, 1.15)	0.82 (0.68, 0.97)	1.17 (0.84, 1.63)	0.93 (0.72, 1.20)	0.92 (0.71, 1.19)
Outer regional area	0.66 (0.46, 0.96)	0.82 (0.67, 1.02)	0.98 (0.65, 1.47)	1.01 (0.75, 1.36)	0.84 (0.61, 1.16)
Remote/very remote	0.76 (0.43, 1.33)	0.61 (0.44, 0.85)	0.87 (0.45, 1.68)	0.59 (0.35, 0.99)	0.85 (0.51, 1.41)
Highest qualification					
Less than Year 12	1	1	1	1	1
Year 12 or equivalent	1.36 (0.96, 1.94)	1.39 (1.11, 1.73)	0.99 (0.65, 1.50)	1.21 (0.88, 1.65)	1.17 (0.85, 1.61)
Trade/Certificate/Diploma	0.91 (0.64, 1.29)	1.10 (0.90, 1.34)	0.94 (0.65, 1.37)	1.00 (0.75, 1.34)	0.80 (0.59, 1.08)
University	1.08 (0.71, 1.65)	1.68 (1.32, 2.15)	0.90 (0.56, 1.46)	1.44 (1.03, 2.02)	1.11 (0.78, 1.58)
Relationship status over time					
Remained not separated/divorced	1	1	1	1	1
Remained separated/divorced	0.58 (0.39, 0.87)	0.14 (0.11, 0.18)	0.86 (0.57, 1.29)	0.29 (0.20, 0.42)	0.88 (0.63, 1.21)
Became not separated/divorced	0.38 (0.23, 0.64)	0.10 (0.08, 0.13)	0.81 (0.51, 1.28)	0.29 (0.19, 0.43)	0.75 (0.52, 1.08)
Became separated/divorced	1.28 (0.95, 1.73)	0.20 (0.16, 0.24)	0.87 (0.60, 1.26)	0.41 (0.30, 0.55)	0.90 (0.67, 1.20)
Stressed about partner for at least half of surveys					
No	1	1	1	1	1
Yes	1.65 (0.98, 2.79)	0.47 (0.31, 0.70)	1.32 (0.70, 2.49)	0.86 (0.48, 1.53)	1.34 (0.78, 2.32)
Stressed about income for at least half of surveys					
No	1	1	1	1	1
Yes	0.88 (0.64, 1.21)	0.37 (0.30, 0.45)	0.94 (0.67, 1.34)	0.63 (0.47, 0.85)	0.84 (0.62, 1.12)
Number of surveys					
6 surveys	1	1	1	1	1
5 surveys	1.48 (1.04, 2.10)	1.00 (0.81, 1.25)	1.47 (1.01, 2.14)	1.22 (0.90, 1.65)	0.89 (0.66, 1.21)
3 or 4 surveys	1.41 (1.05, 1.90)	0.69 (0.57, 0.83)	1.11 (0.79, 1.55)	1.14 (0.88, 1.47)	0.26 (0.18, 0.36)

^a^Multinomial logistic regression with Consistent IPV as reference group (*n* = 821)

The characteristics of women by consistency of reporting IPV over time remained largely the same when only women who responded to all six surveys were examined (Table 4).

**Table 4:** Characteristics of women by consistentcy of reporting IPV for women who responded to all six surveys (*N* = 7100)^a^

Characteristics	Consistent later IPV (OR, 95% CI)	Consistent never IPV (OR, 95% CI)	Mainly IPV (OR, 95% CI)	Mainly no IPV (OR, 95% CI)	Mixed IPV (OR, 95% CI)
Area of residence [ref: Major city]					
Inner regional area	0.87 (0.57, 1.31)	0.86 (0.68, 1.08)	1.37 (0.87, 2.17)	0.86 (0.62, 1.20)	0.98 (0.71, 1.34)
Outer regional area	0.77 (0.45, 1.29)	0.92 (0.69, 1.22)	1.27 (0.72, 2.22)	1.12 (0.76, 1.67)	0.92 (0.62, 1.36)
Remote/very remote	1.12 (0.53, 2.38)	0.64 (0.41, 1.01)	0.98 (0.37, 2.55)	0.64 (0.32, 1.29)	0.92 (0.49, 1.73)
Highest qualification [ref: Less than year 12]					
Year 12 or equivalent	1.24 (0.74, 2.07)	1.29 (0.97, 1.73)	1.19 (0.68, 2.08)	1.04 (0.68, 1.59)	1.24 (0.84, 1.83)
Trade/Certificate/Diploma	1.00 (0.63, 1.59)	1.07 (0.84, 1.38)	1.17 (0.72, 1.90)	1.03 (0.72, 1.49)	0.86 (0.60, 1.24)
University	1.28 (0.76, 2.18)	1.71 (1.26, 2.31)	0.96 (0.51, 1.82)	1.54 (1.01, 2.34)	1.12 (0.74, 1.70)
Relationship over time [ref: Remained not separated/divorced]					
Remained separated/divorced	0.57 (0.32, 1.03)	0.13 (0.10, 0.17)	0.63 (0.35, 1.15)	0.25 (0.15, 0.42)	0.72 (0.48, 1.08)
Became not separated/divorced	0.42 (0.20, 0.87)	0.11 (0.08, 0.15)	0.94 (0.51, 1.74)	0.31 (0.18, 0.53)	0.87 (0.56, 1.34)
Became separated/divorced	1.25 (0.82, 1.90)	0.18 (0.14, 0.23)	0.80 (0.48, 1.32)	0.36 (0.24, 0.54)	0.88 (0.62, 1.25)
Stressed about partner for at least half of surveys [ref: No]					
Yes	3.12 (1.27, 7.68)	0.70 (0.34, 1.44)	2.43 (0.89, 6.68)	1.24 (0.46, 3.37)	1.57 (0.66, 3.72)
Stressed about income for at least half of surveys [ref: No]					
Yes	0.57 (0.33, 0.98)	0.31 (0.23, 0.41)	1.36 (0.85, 2.19)	0.59 (0.38, 0.91)	0.87 (0.60, 1.25)

^a^Multinomial logistic regression with Consistent IPV as reference group (*n* = 470)

## Discussion

This study aimed to examine the consistency of self-reported IPV in a longitudinal cohort survey setting. Across six surveys, 76.9% of women consistently reported never experiencing IPV across any survey that they completed, 10.7% of women consistently reported IPV across all surveys that they completed, with the remaining 12.4% of women inconsistently reporting IPV across surveys. Among the inconsistent IPV responders, two-thirds were consistent most of the time, with 19% of women mainly reporting IPV across surveys, 43% of women mainly reporting no IPV, and 38% of women (4.7% of the total sample) providing mixed IPV responses across surveys. These results suggest that a cross-sectional study may illicit disproportionate false positive and false negative reports of IPV, and that longitudinal data capture is more likely to provide an accurate representation of the population prevalence of IPV. Understanding the factors that influence IPV responding is imperative to the collection of accurate data, which are used to inform prevention and intervention policies.

More than half of the women who indicated that they had experienced IPV at one point in time later said they had never had this experience. These findings build on past research that found similar issues with consistency in regard to self-report of traumatic and abuse events where the perpetrator was not specified [16–18]. The findings suggest that, at least for single item IPV measures, there is a considerable margin for error in ascertaining the lifetime prevalence of IPV. The nature of this error requires further exploration.

There were systematic demographic and health differences between those who responded consistently and those who did not. Women who always reported IPV were similar to women who mainly reported IPV on demographic and health measures, while women who consistently reported never experiencing IPV were similar to women who mainly reported never experiencing IPV. This becomes problematic at the cross-sectional level when examining demographic and health correlates of IPV, due to the risk of IPV misclassification. Given the similarities between groups, it is possible that those who mainly reported IPV represented women who might have provided a single false negative response, thus contributing to under-reporting; while those who mainly reported never experiencing IPV provided a single false positive response, thus contributing to over-reporting.

Past research has indicated that women may under-report IPV due to fear of retribution and feelings of shame or embarrassment [24], which reflects intentional under-reporting. Unintentional under-reporting could occur due to a lack of recognition that experiences constituted violence [25, 26]. Ackerman found that women unintentionally over-reported IPV when behavioural items were interpreted literally [27]. For example, a ‘slap’ might be recorded where the experience was undertaken in jest and not in any way harmful or intimidating. Yet, this scenario would seem unlikely in the current study, where the single item asked only about the experience of living in a ‘violent relationship’. Ackerman did not find evidence for intentional over-reporting among women [27], although some literature proposes that women may over-report to gain favourable outcomes in divorce proceedings [28–30]. Potential gains of providing a false positive response in a confidential survey environment are unknown, as are the motivations. In fact, past research has indicated that women are more likely to respond affirmatively to sensitive questions within a confidential questionnaire than in other settings [14]. It would be useful for future research to determine the degree to which inconsistent responses reflect false positive or false negative responses.

The major limitation of the current paper is the single item used to assess the presence of IPV, which is likely to lead to under-reporting when compared to asking women behavioural questions [31]. However, some screening programs operate on a single question about violence in the home [32–34], so it is important to understand what happens when single questions are asked which require recognition of violence. A further limitation is the age of the sample, all of whom were born 1946–51. It is possible that there are idiosyncrasies particular to women of this age that might not apply to women born in different eras. It should also be noted that women with a tertiary level of education were overrepresented. ALSWH participants from the 1946-51 cohort who were ineligible for the current study were more likely to have reported IPV at baseline than those who were eligible. However, these women were not eligible for the current study, so their propensity for consistent or inconsistent reporting has not been examined. Most of these women took part in the first survey only and do not generally represent those women likely to continue participating in a longitudinal study. One should remain cognisant that the prevalence of IPV may be under-estimated in a longitudinal setting due to participants who may not actively participate in later surveys. With regard to generalisability, the results directly relate to Australian women born 1946–51, within the constraints mentioned above. However, given the timeframe over which IPV has been measured, it would be reasonable to suggest that the results pertain to women in middle to early old age. The results point to the need for future research to establish the existence and nature of reporting IPV among younger women and women in other cultural settings. Finally, we note that this analysis was focused on women, with more research required to understand how other members of the population might respond to items that ask about IPV.

Women who respond inconsistently on a single occasion could be categorised with groups that reflect the majority of their responses (i.e. Mainly IPV with the Consistent IPV group; Mainly never IPV with the Consistent never IPV group). However, categorising women who respond inconsistently on more than one occasion is problematic. More research is required to understand the factors that cause women to respond in a contradictory manner to questions that ask about IPV.

## Conclusion

Our study demonstrates the advantage of longitudinal monitoring of IPV. Relying on a single cross-sectional survey may potentially include around 12% false positive or false negative responses, which are only detectable when IPV is monitored over time. In many countries, the prevalence of IPV is measured at the national level using cross-sectional survey designs. Our results highlight the potential difficulty in obtaining an accurate point prevalence from single surveys, which may be used to inform policy. Nevertheless, it is encouraging that around 88% of women were very consistent when reporting IPV. Even though it is a sensitive topic, confidential longitudinal surveys can obtain a consistent measure of IPV.

## Data Availability

Data are available upon request. The process for accessing the data is documented on the Australian Longitudinal Study on Women’s Health website [http://www.alswh.org.au/how-to-access-the-data/alswh-data].

## Abbreviations

ALSWH: Australian Longitudinal Study on Women’s Health
IPV: Intimate partner violence
